# Therapeutic Perspectives of Thermogenic Adipocytes in Obesity and Related Complications

**DOI:** 10.3390/ijms22137177

**Published:** 2021-07-02

**Authors:** Chih-Hao Wang, Yau-Huei Wei

**Affiliations:** 1 Graduate Institute of Biomedical Sciences, China Medical University, Taichung 406040, Taiwan; chih-hao.wang@cmu.edu.tw; 2 Center for Mitochondrial Medicine and Free Radical Research, Changhua Christian Hospital, Changhua City 50046, Taiwan; 3 Institute of Clinical Medicine, National Yang-Ming University, Taipei 11221, Taiwan

**Keywords:** brown adipose tissue, cell therapy, CRISPR technology, diabetes, gene therapy, thermogenic adipocytes, obesity

## Abstract

There is a rapidly increasing prevalence of obesity and related metabolic disorders such as type 2 diabetes worldwide. White adipose tissue (WAT) stores excess energy, whereas brown and beige adipose tissues consume energy to generate heat in the process of thermogenesis. Adaptive thermogenesis occurs in response to environmental cues as a means of generating heat by dissipating stored chemical energy. Due to its cumulative nature, very small differences in energy expenditure from adaptive thermogenesis can have a significant impact on systemic metabolism over time. Targeting brown adipose tissue (BAT) activation and converting WAT to beige fat as a method to increase energy expenditure is one of the promising strategies to combat obesity. In this review, we discuss the activation of the thermogenic process in response to physiological conditions. We highlight recent advances in harnessing the therapeutic potential of thermogenic adipocytes by genetic, pharmacological and cell-based approaches in the treatment of obesity and metabolic disorders in mice and the human.

## 1. Introduction

Obesity is defined as a chronic disease in which body fat accumulation promotes adipose tissue dysfunction and results in many negative metabolic and psychosocial health consequences [1]. According to the report from the World Health Organization in 2016, the prevalence of obesity and overweightness in adults reaches 39% worldwide and 73.6% in the United States. Unfortunately, obesity is a major risk factor for many diseases, notably type 2 diabetes (T2D), atherosclerosis, hypertension and cancer. These obesity-related diseases have resulted in annual medical costs in the United States reaching up to $147 billion US dollars. Due to the myriad of genetic and environmental factors that are involved in obesity, the therapeutic options that are advised by doctors are few in number. The major advices given to obese and overweight subjects include dietary control and exercise, however, these methods can be very difficult for the subjects and can result in multiple instances of relapse. Moreover, bariatric surgery is an alternative intervention method for obese people by altering gut hormone levels that are responsible for hunger and satiety. This surgery causes significant long-term weight loss, improvements in cardiovascular and diabetic risk factors and a reduction in mortality from 40% to 23% [2]. However, this invasive surgery has been reported to cause adverse effects such as Dumping syndrome, bowel obstruction and complications due to the rapid weight loss. In order to contain this increasingly spreading epidemic, it is beneficial to find therapeutic options that can improve conditions of obese subjects around the world.

There are two types of adipose tissues in the human body including brown adipose tissue (BAT) and white adipose tissue (WAT). BAT is characterized by having multiple, smaller lipid droplets and containing many more mitochondria in comparison to WAT. This allows brown adipocytes to burn energy via thermogenesis. The primary gene product involved in thermogenesis is uncoupling protein 1 (UCP1). UCP1 is exclusively expressed in BAT and works by uncoupling oxidative phosphorylation from mitochondrial respiration, which results in heat production [3]. Additionally, BAT displays a great ability to uptake substantial amounts of circulating free fatty acids and glucose as fuels for thermogenesis, which results in energy consumption within the body [4].

White adipocytes are characterized by a large unilocular lipid droplet that inhibits most of the space in the cell and possesses much fewer mitochondria. White adipocytes are responsible for storing energy within the body in the form of triglycerides and are commonly expanded in obese subjects. Two major types of WAT, which are subcutaneous and visceral white fats, can be found beneath the skin and surrounds the internal organs, respectively. Visceral white fats have been suggested to be associated with the risk of T2D and cardiovascular disease. Subcutaneous white fats are potentially able to be induced to acquire the features and thermogenic functions of BAT, which are termed as beige or brown-like adipocytes. Converting white to beige adipocytes, which is also known as the beiging or browning process, can be stimulated by cold acclimation, exercise training or pharmacologic activation of the β-adrenergic receptors [5]. Two possible models for beige adipocytes formation have been established. Beige adipocytes can form via reprogramming of white mature adipocytes (transdifferentiation) or through de novo differentiation of resident progenitors within the WAT.

Thermogenic adipocytes including brown and beige adipocytes are previously considered non-existent in adult humans. However, this concept was later modified when studies had demonstrated that active thermogenic adipocytes are actually present in adult humans. Using positron emission tomography (PET) and computed tomography (CT) scanning, the areas of thermogenic adipocytes show the significant presence of ^18^F-fluorodeoxyglucose (^18^F-FDG), indicating high glucose uptake [6,7,8]. Additionally, it has been found that cold exposure increases BAT activation and WAT browning in the body, resulting in increased thermogenesis [9].

**Figure 1:**
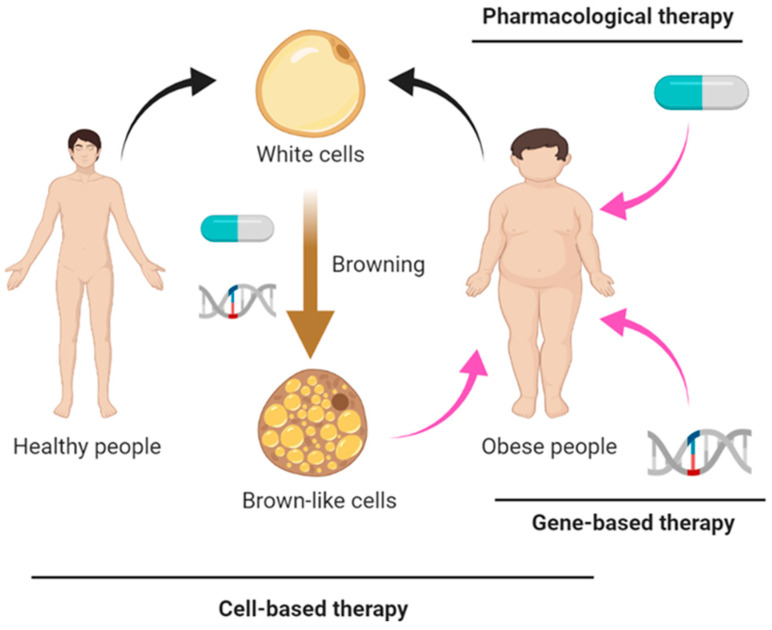
**Cell-based, gene-based and pharmacological therapies to increase thermogenic adipocytes in obese subjects.** In pharmacological therapies, several compounds, proteins, lipids or metabolites have been applied for treatment of obesity through the regulation of thermogenic adipocytes activity and energy expenditure. In gene therapy, the delivery of DNA (transgene), mRNA, microRNA, or Cas9/gRNA system can be used to modulate the expression of genes involved in the thermogenic pathway. Cell-based therapies include autologous and allergenic cell therapies depending on the source of the transplanted cells. Precursor cells isolated from obese subjects (autologous) or healthy donors (allergenic) can be engineered and differentiated to thermogenic adipocytes followed by transplantation of the fat back to the obese subjects.

Given the role of thermogenic adipocytes in energy expenditure and modulation of glucose and lipid metabolism and its crosstalk with other tissues, thermogenic adipocytes have been considered a promising therapeutic target to combat obesity and metabolic disorders. Overweight and obese subjects were found to have decreased thermogenic adipocytes activity in comparison to lean and healthy subjects [8,10]. Several studies have demonstrated that increasing the amount and activity of thermogenic adipocytes improves metabolism in mouse models of obesity or diabetes [11]. Cold-activated or drugs-activated thermogenic adipocytes can also improve insulin sensitivity and glucose tolerance in healthy subjects and patients with T2D [12,13,14]. From the translational point of view, we focus on the therapeutic potential of activating thermogenic function or converting white to thermogenic adipocytes in human subjects or human cells by genetic, pharmacological and cell-based approaches (Figure 1).

## 2. Pharmacological Approaches

Cold is the most potent stimulus to activate BAT function and induces the conversion of WAT to beige fat through stimulating the β adrenergic signaling in both humans and mice. However, cold exposure is inconvenient, uncomfortable and an unrealistic intervention to exert at bedside. In addition, cold may induce other unwanted effects such as high blood pressure [15]. Therefore, much effort has been made in identifying more specific potent pharmacological products that mimic the cold effects on adipose tissues with minimized adverse consequences (Table 1).

### 2.1. Mirabegron

β3-adrenergic receptor (β3-AR) agonists are potent activators of BAT thermogenesis and WAT browning in rodents. However, several attempts to develop β3-AR agonists for application in humans have not yet been successful. Mirabegron (Myrbetriq) is a β3-AR agonist approved by U.S. Food and Drug Administration (FDA) for the treatment of overactive bladder (OAB) by relaxing the bladder muscles and ameliorating the symptoms [28]. Acute single doses of the mirabegron treatment (200 mg) increased BAT glucose uptake and energy expenditure in healthy human subjects [16,17]. However, this treatment exhibited adverse effects such as increased blood pressure, which explains why dosages higher than 50 mg are not clinically used for treatment of an overactive bladder. Blondin et al. recently found that 200 mg of mirabegron activates BAT yet 50 mg does not, although both increases energy expenditure in humans [18]. High dosage may activate human BAT by β2-AR and not β3-AR. Recently, chronic and low doses of the mirabegron treatment has been applied to healthy [13] and obese [14] subjects and results in the activation of thermogenic adipocytes, reduction in inflammatory response in adipose tissues and skeletal muscle, improvements in glucose tolerance, insulin sensitivity and pancreatic β-cell function with little side effects on cardiovascular function [13,14]. Another approach is using a combination of compounds to activate β3-adrenergic signaling and to block β1-adrenergic signaling in order to reduce the cardiovascular dysfunction. Nebivolol is a racemic mixture, which contains D-enantiomers and L-enantiomers. While D-enantiomer acts as a specific β1-adrenergic receptor blocker, L-enantiomer behaves as a β3-adrenergic receptor agonist. Treatment of Nebivolol was shown to induce lipolysis and browning of human white adipocytes through functioning as a β3-adrenergic receptor agonist in vitro [29]. The in vivo effects and side effects of Nebivolol remain to be investigated.

### 2.2. Capsaicinoids

Capsaicinoids include capsaicin, dihydrocapsaicin, nordihydrocapsaicin, homocapsaicin and homodihydrocapsaicin and they are a group of phenolic alkaloids and active components in chili peppers [30]. Interest in the use of capsaicinoids has recently grown due to their anti-obesity effects. Oral supplement of capsaicinoids in overweight or obese subjects induces fat-mass loss by increasing fatty acid oxidation and resting whole-body energy expenditure compared to the placebo-control groups [19,20]. Most importantly, by using the ^18^FDG-PET scan, Yoneshiro et al. showed that dietary supplementation of capsaicinoids enhanced glucose uptake in the BAT region and that activation of BAT was highly correlated with the increase in energy expenditure of the human subject [21]. Capsaicin can also trigger the browning of WAT via upregulation of the expression of UCP1 and BMP8. Capsaicin acts on transient receptor potential cation channel subfamily V member 1 (TRPV1), resulting in the elevation of intracellular levels of Ca^2+^ ions and downstream signaling. TRPV1 deletion completely blocks the capsaicin-mediated anti-obesity effects in mice [31]. In addition, a recent study showed that the combination of capsaicinoids treatment with mild cold exposure (17 °C) synergistically promotes beige adipocyte development and weight loss [32]. These findings have provided a potential therapeutic regimen by combining the dietary and environmental modifications in the treatment of obesity and overweight-related diseases.

### 2.3. Resveratrol

Resveratrol (3,4′,5-trihydroxystilbene, RSV) belongs to a class of polyphenolic compounds called stilbenes. RSV is naturally found in the roots of white hellebore, mulberries, red wine, grapes and peanuts. Administration of RSV was shown to induce beige adipocytes formation in WAT resulting in enhanced glucose uptake and energy consumption in vitro and in vivo [33]. Mechanistically, RSV activates the AMPK–SIRT1–PGC-1α signaling pathway and promotes mitochondrial biogenesis and UCP1 expression. Furthermore, RSV treatment can directly elevate the intracellular cAMP level by inhibition of cyclic AMP (cAMP)-specific phosphodiesterases in WAT to activate cAMP-PKA pathway [34]. Recently, Hui et al. also showed that RSV improves glucose homeostasis in a genetically obese mouse model by BAT activation and WAT browning through a mechanism involving the alteration of gut microbiota [35].

### 2.4. Curcumin

Curcumin, also called diferuloylmethane, is a yellow pigment hydrophobic polyphenol found in the extracts of Turmeric roots, which is a flowering plant of the ginger family. It is best known for its anti-inflammatory function and the ability to increase the antioxidant capacity. Recently, curcumin supplementation has revealed anti-obesity effect in animal models. Lone et al. demonstrated that the dietary curcumin induces browning phenotypes in WAT by the upregulation of the expression of brown fat-specific genes [36]. This effect was mediated through the activation of AMPK singling. In addition to direct effects on white adipocytes, curcumin could also increase the number of the alternatively activated macrophages within WAT to contribute to the browning process [37].

### 2.5. Hormones

It has been established that thyroid hormones (THs) activate thermogenic functions in BAT of mice. Considering the translational implication, studies have tested whether THs or their analogs (levothyroxine and liothyronine) can be used as therapeutics in humans. It was found that treatment of levothyroxine could enhance the basal energy expenditure and glucose uptake of BAT in patients with thyroidectomy (NCT02499471) [22]. In another clinical trial, patients with mutations of insulin receptors who displayed severe insulin resistance were administrated liothyronine for 6 months. This intervention promoted glucose uptake in WAT and muscles in these patients. However, due to the small volume of BAT in human subjects, BAT glucose uptake was not quantified in this clinical trial (NCT02457897) [23].

In light of the controversial results in glucocorticoids (GCs)-mediated BAT activity in rodents and humans, clinical trials have been performed to evaluate the therapeutic roles of GC analogs (hydrocortisone and prednisolone) on BAT function. In a clinical trial applying acute hydrocortisone for 14 h, healthy individuals displayed activation of supraclavicular BAT with higher skin temperature and ^18^F-FDG uptake in both fasting basal conditions and β-AR stimulation as compared with the placebo group (NCT02457897) [24]. In contrast, prolonged GCs treatment possesses opposite outcomes. One week of oral prednisolone treatment showed reduced BAT activity in response to cold exposure or meal stimulation (NCT02457897) [25]. The discrepancy of the BAT activity between the above-mentioned studies may result from the indirect or compensatory effects in the prolonged treatment.

### 2.6. GLP1 Receptor Agonists

Glucagon-like peptide 1 (GLP-1) was found to activate BAT thermogenesis in mice [38,39], whereas it seems to have opposite effects in humans. Flint et al. reported that GLP-1 infusion decreased diet-induced thermogenesis due to the reduction in food absorption and limiting the nutrient supply in healthy men [26]. Another study demonstrated that subcutaneous injection of exenatide, a GLP-1 receptor agonist, dramatically decreased energy intake with no change in the resting energy expenditure in obese subjects (NCT00856609) [27]. Considering the profound effects of GLP-1 on gut and other systemic effects, an ongoing clinical trial aims to investigate the specific effect of SAR425899, which is a novel GLP-1 agonist, through direct injection to the subcutaneous WAT of obese subjects (NCT03376802).

### 2.7. Proteins

Bone morphogenetic protein 7 (BMP7) promotes the commitment of mesenchymal progenitor cells to a brown adipocyte lineage and plays a crucial role in embryonic brown fat development in mice [40]. In two studies, BMP7 was administered during the differentiation of human adipose-derived stem cells (hASCs) isolated from either superficial neck fat [41] or abdominal subcutaneous fat to induce the thermogenic gene program [42]. The results indicate that BMP7 can drive human adipogenic stem cells to differentiate into metabolically active beige adipocytes.

Fibroblast growth factor 21 (FGF21) administration in mice was found to improve insulin-stimulated glucose uptake and activate thermogenesis in brown/beige adipocytes [43]. Similar to the effects observed in mice, FGF21 analogs (PF-05231023 and LY2405319) decreased body weight, body fat and serum lipid content in obese monkeys and humans, respectively [44,45]. However, the small glucose lowering effect of FGF21 in the human subjects could be explained by lower amount of BAT in humans compared to mice.

## 3. Gene-Based Therapy

Certain genes have been targeted to activate BAT function and promote the browning of WAT. There is no doubt that the activation of UCP1-mediated thermogenesis is an efficient method to dissipate excess energy and consume fuels to provide metabolic health benefits [46]. Gene-based therapy is an intervention by which genetic materials are introduced into the target cells/tissues to permanently manipulate gene expression and treat diseases. Genetic manipulation in WAT to enhance energy expenditure and fatty acid/glucose utilization through a “browning” process, which provides a potential therapeutic approach to combat obesity and T2D, has been elucidated in mice [11]. However, only few studies have applied this therapeutic strategy to human cells in vitro so far and none have been directly applied to the human subjects yet. Here we review the genetic manipulations performed in human adipocytes or stem cells, which are potentially applicable to cell-based therapy or direct gene therapy in the human (Table 2), and discuss the translational perspectives for the future.

### 3.1. Virus-Based Gene Therapy

Mice that ectopically expressed UCP1 in skeletal muscle [47,48] or adipose tissue [49,50] displayed increased energy expenditure and were protected from diet-induced obesity. Several studies have demonstrated that overexpression of UCP1 enables the uncoupling of respiration and mitochondrial biogenesis in non-thermogenic human cells [59,60]. Introduction of the upstream transcriptional regulators such as PRDM16 and PGC-1α induces UCP1 transcription and drives conversion of WAT to beige fat and increases energy expenditure in mice [11]. Tiraby et al. [51] isolated and differentiated human preadipocytes from the subcutaneous abdominal white adipose tissue and delivered human PGC-1α transgene into mature adipocytes using adenoviral infection. The engineered adipocytes were able to acquire the brown-like phenotypes including the expression of UCP1 and enzymes/proteins involved in β-oxidation of fatty acids and mitochondrial respiration [51]. In addition, introducing PRDM16 and C/EBP-β transgenes together into human inducible pluripotent stem cells (iPSCs) using retroviral infection induced the formation of lipid-laden brown adipocytes with an increase in mitochondrial function and oxidative metabolism [52]. Furthermore, human dermal fibroblasts were directly converted to brown adipocytes with a robust elevation of thermogenic function by the overexpression of transcription factors c-MYC and C/EBP-β [52]. Although engineering fibroblasts to human brown adipocytes provides a more convenient and more potent system than using iPSCs, the safety issue should be considered when using the oncogene of c-MYC. Moreover, adenoviral overexpression of KLF11, which is a co-activator for PPARγ super-enhancer formation, has also been successfully applied to reprogram human white adipocytes to beige adipocytes [53].

Natural serotypes of adeno-associated virus (AAV) can be used for specific delivery to liver and muscle, but not for WAT and BAT. Recent efforts have focused on engineering the AAV vectors to create novel recombinant AAVs (rAAV) with diverse characteristics including specific targeting and low immunogenicity. Huang et al. engineered a hybrid serotype of rAAV, Rec2. After oral administration, Rec2 rAAV tended to preferentially target BAT with higher efficiency and specificity compared to the other natural serotypes of AAVs. The Rec2 rAAV successfully and specifically delivered the VEGF transgene to BAT and promoted thermogenesis. The finding suggested that engineered AAVs, such as Rec2, may provide a potent and specific delivery system to manipulate BAT for therapeutic applications [61].

### 3.2. miRNAs

MicroRNAs (miRNAs) control gene expression by decreasing the mRNA stability or inhibition of the translation of mRNA. Recently, miRNAs-based therapy has been successfully applied to treat several diseases [62]. The regulation of thermogenic adipocyte differentiation and function by miRNAs has been elucidated [63]. In terms of using miRNA-based therapeutics in human cells, inhibition of miR-27 during the adipogenic differentiation of human adipose-derived stem cells promotes the upregulation of many key transcription factors including PPARγ, PRDM16 and PGC1α, which in turn enables the induction of UCP1 and mitochondrial biogenesis [54]. Given the low efficiency of non-viral-based gene delivery in mature adipocytes, Isidor et al. developed a more efficient small interfering RNA (siRNA) reverse transfection protocol for delivering a desired gene into mature adipocytes. Introduction of siRNA targeting the negative regulators of the browning process can promote the thermogenic program and convert human white adipocytes to brown adipocytes with a rather high efficiency [64].

### 3.3. CRISPR/Cas9-Based Gene Therapy

The CRISPR/Cas9 system presents a powerful strategy for genome editing in mammalian cells [65]. Several new tools have been developed based on CRISPR/Cas9 that allows targeted inhibition or activation of gene expression or inserting a transgene into the genomic DNA. One study takes advantage of the CRISPR/Cas method to replace a truncated UCP1 gene with a UCP1 transgene driven by the adiponectin promoter into endogenous loci in pigs. Pigs with the reconstituted UCP1 gene became leaner and displayed enhanced cold tolerance [55]. Recently, Shen et al. [56] developed CRISPR-delivery particles (CriPs), which are Cas9/sgRNA RNP complex coated with amphiphilic peptides, to allow more efficient gene editing in primary white preadipocytes [56]. CriPs-mediated deletion of nuclear receptor-interacting protein 1 (Nrip1), which is also known as the receptor-interacting protein 140 (Rip140), successfully promoted browning of white adipocytes in vitro. Nrip1/RIP140 is a co-repressor known to negatively regulate UCP1 expression. However, further in vivo investigation is required to evaluate the therapeutic potential of this strategy.

For the improvement of the CRISPR activation techniques, a nuclease-deactivated Cas9 (dCas9) is fused with transactivation domains and directed by a single guide RNA (sgRNA) targeting a specific promoter where this synthetic transcriptional complex can drive the expression of the endogenous gene [66]. This approach has been used to activate the brown/beige adipocyte genes (Prdm16, Zfp423 and Ucp1) in mouse white preadipocytes [57]. Recently, CRISPR-mediated gene activation has been achieved by direct injection of viruses containing these constructs into tissues to ameliorate the progression of diseases in mouse models, including those of type I diabetes, acute kidney injury and muscular dystrophy, respectively [67].

Delivery of ribonucleoprotein (RNP) for the gene therapy, especially in CRISPR/Cas9 genome editing, offers several advantages over viral delivery. Unlike the uncontrolled transgene integration and prolonged expression caused by using viral delivery, RNP complex is rapidly degraded and therefore enables transient genetic manipulation, limiting the off-target effect and reducing the immune response. Considering that the transcriptional and translational machineries in some cell types are not compatible with expressing the transgene driven by certain promoters, introducing the RNP complexes enables the immediate gene editing and skips the transcription or translation of Cas9 and sgRNA [68]. Recently, Yin et al. succeeded in the delivery of non-viral Cas9 (Cas9 mRNA and sgRNA) using lipid nanoparticle formulations [69]. Importantly, such LNP-mediated gene therapy has been evaluated in clinical trials or applied in FDA-approved RNAi drug (Patisiran) [70]. This established or approved delivery system in clinical use would accelerate future applications for targeting BAT activation genes in the human.

## 4. Cell-Based Therapy

Cell-based therapies offer treatment strategies for patients with diseases that the existing pharmaceuticals cannot adequately address. One potential benefit of a cell-based approach, compared to strategies based on single molecules, is that the cells serve as a “living factory” to provide a more comprehensive and persistent therapeutic effect. Autologous cell therapy is a preferred therapeutic intervention in which cells are taken from an individual and administered into the same person to minimize the immune rejection. Autologous cell-based therapies have been an active area of research and are moving towards commercial development and patient access partially due to the recent advancements in the delivery systems and genome engineering methods such as CRISPR/Cas9 [69,71]. Here we discuss the therapeutic strategies using tissue/cell transplantation to increase the number of thermogenic adipocytes in mice with obesity or related metabolic disorders (Table 3) and their potential for applications in the human.

### 4.1. Tissue Transplantation

Given the role of BAT as a secretory organ that communicates with other tissues to modulate systemic metabolism, the interest has grown in exploring BAT transplantation to provide therapeutic benefits in patients with metabolic disorders. In type 1 diabetes (T1D), transplantation of insulin-secreting β-cells to restore the insulin level is commonly used. In addition to the dysfunction of pancreatic cells, T1D patients also suffer from the loss of adipose tissues. Embryonic BAT transplantation in the subcutaneous space of STZ-induced T1D mice significantly improves glycemic control, normalizes glucose tolerance and reduces tissue inflammation. The effect has been proposed to be mediated by insulin-like growth factor 1 (IGF1) secretion from transplanted BAT [72,73].

Other studies demonstrated that the transplantation of adult BAT into the dorsal subcutaneous region of diet-induced obese [74] or genetic obese (ob/ob) [77] mice improved metabolic phenotypes including reduced weight gain, increased energy expenditure, enhanced glucose tolerance and insulin sensitivity and reversed the secreted adipokine profile. Most interestingly, transplanted BAT seemed to promote the activity of endogenous heart, liver, muscle, WAT and BAT in recipient mice or at least in part by increasing the sympathetic tone [75] or putative factors secreted by BAT [76,78]. In addition to transplanting BAT into the subcutaneous dorsal site, Stanford et al. [76] reported that BAT transplanted into the visceral cavity can survive up to 12 weeks and promotes insulin-stimulated glucose uptake in endogenous WAT and BAT, which is mainly driven by IL-6. Furthermore, transplantation of beige adipose tissue, which was isolated from the mice after exercise training also provided metabolic benefits in HFD-fed recipient mice [78], at least partially mediated by the secretion of TGF-β2.

### 4.2. Cell Transplantation

In clinical applications, the unlimited resource of cells provides stem cell therapy a more feasible strategy than tissue transplantation. Considering the limited amount of BAT and brown adipocyte progenitor cells in the adult human, the approach using white adipocyte progenitors or even less committed stem cells seem more applicable.

Overexpression of PRDM16 and C/EBPβ, which are two important factors involved in thermogenic differentiation, stimulated fibroblasts to differentiate into adipocytes and forms an ectopic fat pad with brown fat-like characteristics after transplanting into mice. These differentiated brown-like adipocytes have been shown to be functional and able to uptake glucose [79]. Alternatively, other groups have used pharmacological treatments to induce thermogenic differentiation in stem cells. Nishio et al. [80] established a method to efficiently differentiate human pluripotent stem cells to brown adipocytes using a hemopoietin cocktail, which is composed of KIT ligand (KITLG), fms-related tyrosine kinase 3 ligand (FLT3LG), IL6, VEGF and BMP7. Upon subcutaneous transplantation into mice, these brown-like adipocytes were stimulated with β-adrenergic receptor agonists and exhibited behaviors similar to endogenous classic brown adipocytes. More recently, Min et al. [81] isolated adipocyte progenitors residing in the capillary network of human WAT and defined an in vitro protocol using adipogenic cocktail and forskolin to differentiate them into beige adipocytes. After mixing with Matrigel and implanting them into the subcutaneous dorsal region of immunodeficient NOD-SCID IL2reg-null (NSG) mice, human beige adipocytes were able to enhance glucose utilization and improve glucose tolerance in HFD-fed NSG mice [81]. More recently, our group applied a CRISPR synergistic activator to establish human brown-like (HUMBLE) adipocytes via overexpressing UCP1 in human white adipocytes. HUMBLE cells acquired brown fat-like features and provided prolonged metabolic benefits in HFD-fed nude mice after subcutaneous transplantation [58,82].

It is worth noting that different transplantation studies have identified distinct secreted factors mediating the activation of endogenous tissues and the modulation of systemic metabolism. The differences can be attributed to the use of different species (mouse or human), different transplants (whole tissue, beige cells or brown-like cells) and different transplantation sites (visceral cavity or subcutaneous site). Each study examined a unique condition that generated certain signals contributing to the wide range of effects and phenotypes reported.

In elderly subjects or lipodystrophy patients, the number and the differentiation capacity of adipocyte progenitors and stem cells are limited [83]. It is difficult to expand and differentiate them into enough brown/beige adipocytes to allow autologous cell transplantation. In these cases, progenitor cells derived from healthy individuals provide an alternative materials source for allogenic cell transplantation. Strategies to minimize the immune rejection should be considered in allogenic transplantation. Hydrogel-mediated encapsulation has been applied to minimize the immune response, maintain the transplant-host communication and provide a 3D culture environment for transplanted cells [84]. Moreover, engineering of iPSCs to generate hypoimmunogenic iPSCs followed by differentiation and transplantation can enable the immune escape of cells in immunocompetent allogeneic recipients [85].

## 5. Conclusions and Future Perspectives

Thermogenic adipocytes, which include brown and beige adipocytes, arise from distinct origins during embryonic development; however, they share similar effects in thermoregulation and nutrients utilization. Secreted factors from brown/beige adipocytes regulate the function of adipocytes and other cell types could control systemic metabolism through the whole body. This review has focused on the potential of thermogenic adipocytes in the prevention and treatment of obesity, diabetes and related metabolic disorders. We have discussed the advantages and disadvantages of different approaches including genetic, pharmacological and cell-based strategies in the activation or increase in thermogenic adipocytes.

Although multiple therapeutic strategies targeting brown/beige fat have been shown to work well in the rodent models, most of these tools are not applicable to humans. There is a significant knowledge gap in translational application from mouse to the human, especially considering the differences in brown fat between the two species. For example, Blondin et al. [18] recently found that thermogenesis in human brown fat is mainly driven by β2-AR and not by β3-AR, which is the dominant isoform in the adipose tissue of mice. In addition, in order to avoid unwanted side effects of pharmacological therapy on other tissues, targeted delivery of drugs to adipose tissues would offer a promising solution.

In order to mimic human conditions in mice, de Jong et al. recently performed studies in middle-aged mice housed in a thermoneutral condition (30 °C) and fed them with a 45% high-fat diet and concluded that human BAT is similar to murine classical brown fat in terms of cellular, molecular biological and morphological characteristics [86]. Although this manipulation aimed to create a “humanized BAT” in mice, many differences still existed compared to real human BAT, including the cellular heterogeneity and composition within BAT and the developmental origins of BAT between mice and the human [87,88]. In addition, considering the complexity and crosstalk between different cell types within brown/beige fat, using human adipose organoids as a platform to develop a therapeutic strategy may shorten the gaps of translational medicine.

Regarding therapeutic approaches aiming to increase the amount or activity of thermogenic adipocytes, cell-based and gene therapies also offer feasible therapeutic options. Autologous cell therapy is considered a safe and less invasive approach. Allergenic cell therapy can provide a universal cell source when the precursor cells derived from the patient is limited; however, the rejection problem should be solved. Gene therapy using the viral delivery system has been applied in many non-metabolic diseases due to its high efficacy. Importantly, unintended genome integration, high immunogenicity and safety issues associated with gene delivery need to be addressed. Other non-insertional genetic approaches, such as microRNA-based or mRNA-based medicine, which possess a low risk of permanent genomic alteration, may be more applicable in humans. Recent advances in mRNA modification and the delivery system could greatly improve the clinical application. In addition, synthetic mRNA encapsulated with biodegradable lipid nanoparticles was shown to enhance the stability and translatability of mRNA and was successfully applied to the treatment of a genetic disorder in the liver [89]. However, the compatibility of these approaches for targeting brown and white adipose fats needs to be further examined.

In the development of therapeutics using both genetic or pharmacological approaches, tissue-specifc and cell type-specific targeting strategies are greatly desired. Improving targeting specificity enables the reduction in effective dosages and off-target effects and results in cost-effectiveness and safety of the treatment. Customization of gene expression with the cell-specific or inducible promoters renders it possible for selective genetic manipulation in a spatio-temporal manner. Another strategy is to engineer the designated surface receptors or ligands on nanoparticles, carriers or Cas9 protein to be selectively delivered to certain cells via specific binding. In order to specifically target WAT, two adipose vasculature targeting peptides, which are iRGD and P3, have been conjugated to the membrane of nanoparticles. iRGD and P3 bind to integrin αvβ3/β5 receptors and prohibitin, respectively [90].

Moreover, genetic manipulation needs to be strictly controlled in spatial and temporal manners. Recently, Chen et al. applied near-infrared optogenetics and Au nanorod in CRISPR/Cas9 system to regulate gene editing with high precision and spatial specificity. In their design, Cas9 expression is driven by a heat-inducible promoter and is encapsulated by a cationic polymer-coated Au nanorod. Au nanorod not only serves as a carrier to efficiently deliver the plasmid but also works as a photothermal transducer to produce heat for Cas9 expression after receiving the photonic energy from the near-infrared light. Fine-tuning of the optogenetic system offers the spatial and temporal controls required for CRISPR/Cas9-based gene therapy [91].

In conclusion, by combining the current advances in the fundamental body of knowledge and new technologies, harnessing the therapeutic potential of thermogenic adipocytes for combating obesity and related metabolic disease proves to be promising. 

## Figures and Tables

**Table 1:** Activation of thermogenic adipocytes by pharmacological therapies.

Compounds	Populations	Effects	References
Mirabegron	Healthy male subjects	Higher BAT activity Increased EE	[16,17]
	Healthy male subjects	Higher BAT activity at a high dose	[18]
	Healthy women subjects	Higher BAT activity Increased EE	[13]
	Obese subjects	Activated conversion of WAT to beige fat Increase in insulin sensitivity and β cell function	[14]
Capsinoids	Obese subjects	Increased EE	[19]
	Obese subjects	Increased fatty acid oxidation No change in EE	[20]
	Healthy male subjects	Higher BAT activity Increased EE	[21]
Levothyroxine	Patients with thyroidectomy	Higher BAT activity Increased EE	[22]
Liothyronine	Patients with insulin receptor mutation	Increased glucose disposal	[23]
Hydrocortisone	Healthy male subjects	Increased body temperature	[24]
Prednisolone	Healthy subjects	Lower BAT activity	[25]
Synthetic human GLP-1	Healthy male subjects	Decreased EE	[26]
Exenatide (a GLP-1 analog)	Non-diabetic obese subjects	Decrease in body weight and food intake No change in EE	[27]

Abbreviations: BAT, brown adipose tissue; EE, energy expenditure; GLP-1, glucagon-like peptide 1; WAT, white adipose tissue.

**Table 2:** Generation of thermogenic adipocytes by gene-based strategies in mice and humans.

Strategies	Targets	References
Ucp1 OE	Mouse skeletal muscle	[47,48]
	Mouse adipose tissues	[49,50]
Prdm16 OE	Mouse WAT	[11]
PGC-1α OE	Mouse WAT	[11]
	Human mature white adipocytes	[51]
Prdm16 and C/EBP-β OE	Human iPSCs	[52]
c-MYC and C/EBP-β OE	Human dermal fibroblasts	[52]
KLF11 OE	Human mature white adipocytes	[53]
MiR-27 inhibition	Human adipose-derived stem cells	[54]
CRISPR-based Ucp1 reconstitution	Pig WAT	[55]
CRISPR-based Nrip1 deletion	Mouse primary white preadipocytes	[56]
CRISPR-based Ucp1 activation	Mouse white preadipocytes	[57]
	Human white preadipocytes	[58]

Abbreviations: iPSCs, inducible pluripotent stem cells.; Nrip1, nuclear receptor-interacting protein 1; OE, overexpression; Prdm16, PR-domain containing 16 protein; Ucp1, uncoupling protein 1; WAT, white adipose tissue.

**Table 3:** Studies showing increase in thermogenic adipocytes using tissue and cell transplantation in obese mice.

Strategies	Targets	References
**Tissue transplantation**		
Embryonic BAT	STZ-induced T1D mice	[72,73]
Adult BAT	DIO mice	[74,75,76]
	Genetic obese mice	[77]
Exercise-induced beige fat	DIO mice	[78]
**Cell transplantation**		
Gene-induced mouse brown adipocytes	Nude mice	[79]
Drug-induced human brown adipocytes	NOG mice	[80]
Drug-induced human beige adipocytes	DIO NSG mice	[81]
CRISPR-engineered human brown-like adipocytes	DIO nude mice	[58]

Abbreviations: BAT, brown adipose tissue; STZ, Streptozocin; T1D, type 1 diabetes; DIO, diet-induced obesity; WAT, white adipose tissue; NSG and NOG, immunodeficient mice.

## Data Availability

No new data were created or analyzed in this study. Data sharing is not applicable to this article.
